# Mucinous Histology, *BRCA1/2* Mutations, and Elevated Tumor Mutational Burden in Colorectal Cancer

**DOI:** 10.1155/2020/6421205

**Published:** 2020-04-25

**Authors:** Noa Harpaz, Yair Eli Gatt, Roy Zvi Granit, Hila Fruchtman, Ayala Hubert, Albert Grinshpun

**Affiliations:** ^1^Sharett Institute of Oncology, Hadassah-Hebrew University Medical Center, Jerusalem, Israel; ^2^Department of Microbiology and Molecular Genetics, Institute for Medical Research Israel-Canada, Faculty of Medicine, The Hebrew University of Jerusalem, Jerusalem, Israel; ^3^Radiology Department, Hadassah-Hebrew University Medical Center, Jerusalem, Israel

## Abstract

Mucinous colorectal carcinomas (MC) constitute 10% of colorectal malignancies. Recently, an increased risk of colorectal cancer has been demonstrated in germline *BRCA1/2* mutation carriers. Furthermore, *BRCA1/2* germline mutation carriers have exhibited a higher-than-expected frequency of MC tumors. Here, we investigate the relationship between *BRCA* mutations and mucinous histology in colorectal carcinoma patients, using both an existing cohort of sequenced colorectal tumors and a prospective case-control study comparing MC and conventional adenocarcinoma (AC) patients tested for *BRCA* mutations. We discovered that MC tumors exhibit a statistically significantly higher incidence of *BRCA* mutations in addition to a higher average mutation count when compared to AC tumors in the existing cohort. The strongest predictor of the mutation count was mucinous histology, independently of other variables including microsatellite instability. Contrary to our hypothesis, the first association did not recur in the prospective case-control study, likely due to our pathological definition of MC tumors and small sample size. Finally, we observed a higher tumor mutational burden (TMB) in MC tumors compared with AC tumors. We suggest that the association between MC histology, *BRCA* mutations, and increased TMB may open the door to the utilization of simple tests (such as histopathologic characterization) to detect patients who may benefit from immunotherapy in colorectal cancer.

## 1. Introduction

Colorectal cancer (CRC) is the third most common malignancy worldwide, currently accounting for 700,000 deaths worldwide per year. The global burden of CRC, according to recent estimations, is anticipated to rise by 60% by 2030 [1].

While colorectal tumors were previously considered to be a single homogenous entity, it is now known that they are in fact a heterogeneous collection of tumors, each with its own distinct histological and molecular features that vary in their treatment and prognosis. The heterogeneous population of CRC is mainly comprised of two histological subtypes: 10–15% mucinous carcinomas (MC) and 85–90% adenocarcinomas (AC) [2].

MC tumors have a tendency to develop in young patients and are associated with late diagnosis at advanced stages, possibly because their typical location in the proximal colon is associated with less symptomatic presentation and a faster disease progression [3]. Clinically, MC prognosis has proven to be slightly worse than AC, with 2–8% increased hazard of death even when corrected for stage at presentation [4]. A limited response to systemic therapy in metastatic disease has also been reported [5]. MC histology has therefore been considered as an unfavorable prognostic indicator of CRC. This consensus has been recently challenged due to the identification of the importance of the sidedness (right vs. left colon) in the prognosis. This has led to an understanding that for colonic MC tumors there is no difference in overall survival after correction for stage and sidedness [6]. Yet, for rectal MC tumors, there is a reduced rate of complete response and tumor downstaging following neoadjuvant chemoradiotherapy [7].

The carcinogenesis of MC is not clearly understood, though the higher prevalence of MC in hereditary and acquired conditions such as inflammatory bowel diseases, hereditary nonpolyposis colorectal cancer (HNPCC), and past radiotherapy treatment suggests that MC may derive from an alternative oncogenic pathway [8]. Regarding the genetic and molecular patterns, MC tumors tend to overexpress the *MUC2* and *MUC5AC* genes which are responsible for the formation of excess mucous. Other common molecular aberrations in MC include higher incidence of *PI3K*, *SMAD4*, and *BRAF* mutations[5, 9–12]. Importantly, MC tumors are associated with microsatellite instability (MSI), which is known to be involved in most cases of HNPCC and in 15% of sporadic CRCs. MSI is caused by inactivation of DNA mismatch repair genes (e.g., *MLH1* and *MSH2*), triggering an uncontrolled tumor growth [5, 13–15].

Classically, *BRCA1/2* genes encode important proteins responsible for maintenance of genome integrity and response to DNA damage [15, 16]. Hereditary mutated *BRCA1/2* tumor suppressor genes are key factors for pathogenesis and development of breast and ovarian cancers. *BRCA1/2* role in the carcinogenesis of CRC is currently unknown. Recent retrospective study of *BRCA1/2* carriers who developed CRC detected a higher-than-expected incidence of left-sided MC tumors [17]. Ending long-lasting debate, a new meta-analysis has clearly shown a statistically significant increased risk of colorectal cancer development in carriers of *BRCA1* mutations [18].

In this study, we aim to further investigate the relationship between BRCA mutations and mucinous histology in colorectal cancer patients.

## 2. Methods

### 2.1. Patients

Patients were eligible if they were 18 years of age or older and had a colorectal malignancy with valid histology of adenocarcinoma or mucinous features. Patients were considered as MC if the tumor pathology was described as having one of the following features: mucin-producing cells, signet ring cells, a focal mucinous component, or a mucin predominant feature. All patients provided written informed consent for any genetic research. The study was approved by the Institutional Review Board.

Excluded patients were those who did not have available pathology slides or a sufficient quality of material for BRCA analysis.

### 2.2. Database Analysis

A cohort of targeted sequencing of 1134 metastatic colorectal cancer (MSKCC [19]) was accessed via cBioPortal (https://www.cbioportal.org) for analysis. Patients were considered MC if their tumor exhibited one of the following features: mucinous carcinoma, signet ring cells, and a mucinous component. Patients were considered AC if their diagnosis was a conventional adenocarcinoma.

### 2.3. Study Design

#### 2.3.1. Prospective Study Measurements

A prospective case-control study was conducted based on a large academic hospital's cancer center between January 2017 and August 2019 (Hadassah Medical Center). CRC patients with mucinous histology were recruited, along with conventional adenocarcinoma histology controls. Clinical and pathological data were extracted from digital records. Genetic data was analyzed and validated by the pathology department in Hadassah Medical Center or Foundation Medicine tests. Mismatch repair (MMR) status was evaluated by immunostaining for the mismatch repair proteins hMLH1, hPMS2, hMSH6, and hMSH2. Next-generation sequencing tests were conducted to identify alternations in hotspot regions in a few key factor functioning genes by Ion Torrent system. For library construction of *KRAS*, *BRAF*, and *PI3K* genes, Oncomine^TM^ Solid Tumour DNA Kit was used; for *BRCA1/2* genes, Ion AmpliSeq™ Oncomine BRCA primers were used.

Tumor mutation burden (TMB) results were based on either (1) commercial kits (such as 324-gene panel assay FoundationOne® CDx test, validated comparing to whole-exome sequencing (WES) [20]) or (2) local analysis by Pathology Department with Ion Torrent system sequencing and assessed by the Oncomine Tumor Mutation Load Assay (Thermo Fisher Cat. No. A37910), also validated comparing to WES [21].

### 2.4. Database Analysis of TCGA Measurements

It is important to mention that TMB assessed by WES is usually reported as the total number of mutations per tumor, while TMB outputs from gene panel assays are usually normalized to mutations per megabase (mut/Mb) because they differ in the number of genes and target region size [20].

In our paper, we utilize a measure called “mutation count,” defined as somatic nonsynonymous variants in encoding genes by exome sequencing as determined by TCGA [19, 20].

An additional measure we utilize is the MSI score. This measure was also derived from the TCGA database and is evaluated by MSIsensor, a software tool that quantifies MSI in paired tumor-normal genome sequencing data and reports the somatic status of corresponding microsatellite sites in the human genome [22].

### 2.5. Statistical Analysis

In order to compare different variables between the two groups, we used the chi-squared test and Fisher's exact test for categorical variables and the Student *t*-test and Mann–Whitney *U* test for quantitative variables. Analysis of more than two groups was conducted by the Kruskal–Wallis test for quantitative variables and by the chi-squared test for categorical variables. Spearman's rank-order correlation was used to compare two quantitative variables. In the MSKCC cohort, a linear regression model was constructed for all variables that were statistically significantly linked to the mutation count. All *p* values are corrected for multiple hypotheses by the Bonferroni method [23].

## 3. Results

### 3.1. BRCA Mutations Are Linked to MC Histology and a Higher Mutation Count in an Existing Database

To assess whether there is a higher incidence of *BRCA1/2* mutations in MC tumors than in AC tumors, we performed an analysis of a cohort of targeted sequencing of 1134 metastatic colorectal cancer samples [19] (hereby the MSKCC database). The database included 128 MC patients and 725 AC patients (conventional adenocarcinoma), while other histological subtypes were excluded. Our analysis showed a significantly higher incidence of *BRCA* mutations in the MC tumors compared to AC (19/128 MC 14.8%, 30/725 AC 4.1%, *p* value <0.001, by chi-squared). The MSKCC database also includes the mutation count for each sample, defined as somatic nonsynonymous variants in encoding genes by exome sequencing as determined by TCGA; this feature is known to be prominent among MC tumors and is often linked to MSI [24]. Interestingly, several other variables in the MSKCC database presented a similar behavior, several of which were known features of MC tumors (Table 1): age at diagnosis, fraction of genome altered, and primary tumor location (average mutation count for right colon tumor was 20.1 versus 9.5 for left colon tumors). For the latter variable, this relation remained even when examining the exact tumor site (cecum—22, ascending colon—19, hepatic flexure—18.6, and no specific location in right colon—19.5 average mutation count). As expected, the MSI score (see Methods) was also statistically significantly correlated with mutation count (*p* value <0.001).

The average mutation count in tumors with MC histology was 24.8, indeed much larger than the average mutation count of 8.9 for tumors with AC conventional histology (*p* value <0.001). We noticed that *BRCA* mutations were linked to a higher mutation count in a statistically significant manner. We found a much larger amount of mutations in patients with mutated *BRCA* somatic genotypes versus patients with the wild-type (WT) somatic *BRCA* genotype (average of 59.4 versus 9.4, respectively, *p* value <0.001).

### 3.2. *BRCA*-Mutated Tumors Can Be Divided into a High Mutation Count Group with Mucinous Histology and a Low Mutation Count Group with Adenocarcinoma Histology

**Figure 1:**
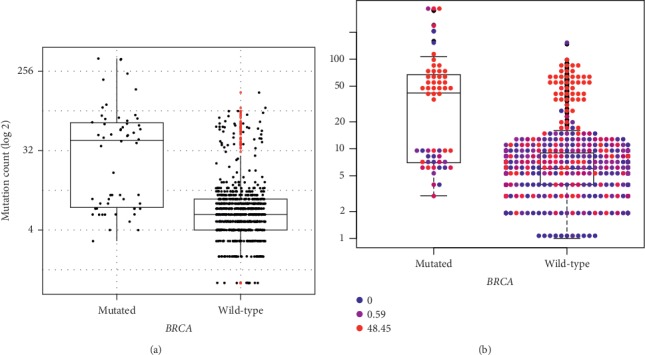
Relationship between *BRCA*-mutated vs. WT tumors and overall mutation count (a). Boxplot-swarmplot with the individual swarmplot points colored by MSI (b). MSI level score is calculated by MSIsensor (Niu et al. [25]) from blue (0) to red (48.45). Both plots are log-scaled.

While tumors with *BRCA* mutations indeed tended to have higher mutation counts (Figure 1(a)), the analysis revealed two distinct groups of *BRCA*-mutated tumors that differ significantly in their mutation count: a group with high mutation count and group with low mutation count. While some of the variability between these two groups could be explained by MSI score, some of the *BRCA*-mutated tumors did not have a high MSI score despite a high mutation count (Figure 1(b)). We decided to employ two parallel strategies in order to further explore this phenomenon. (A) We compared the different variables in the MSK database between the two groups. (B) We studied the relationship of the different variables with the mutation count directly among *BRCA*-mutated tumors. We suspected that some features would discriminate between the two groups, and, indeed, fraction of genome altered, tumor sample histology, stage at diagnosis, primary tumor locations, and MSI score were significantly different between the two groups (Table 2), a result that was in complete agreement between the two strategies we employed. Finally, we constructed a linear regression model for the mutation count among *BRCA*-mutated tumors, utilizing the features found to be statistically significant in the previous analysis (Table 3). Nearly 0.40 of the variances in the mutation count between the different patients with *BRCA* mutations could be explained using these variables alone. The strongest predictor of the mutation count was mucinous histology, independently of other variables.

### 3.3. A Prospective Cohort Questions the Relationship between BRCA Mutations and Histological Features

**Figure 2:**
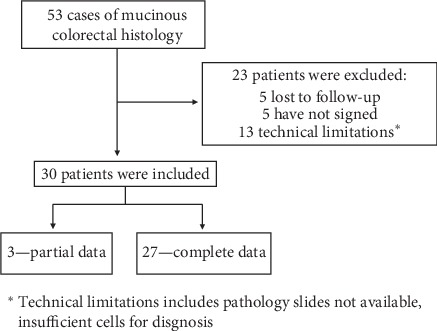
Prospective trial enrollment of patients with mucinous colorectal cancer.

At our cancer center, we prospectively enrolled 93 CRC patients, 53 cases of patients with MC tumors and 40 with AC tumors. Of 53 MC patients, 30 were included (Figure 2). None of the background features differed significantly between the mucinous histology and adenocarcinoma histology groups, indicating that the two groups were not biased by their background properties (Table 4). Since *KRAS*, *BRAF*, and *PI3K* mutations are known to have a higher frequency in MC patients [5, 9–12], we performed sequencing tests for those mutations. However, we found no statistically significant differences in the frequencies of these mutations between the two groups, though there was a positive trend in the *KRAS* mutations towards MC group (*p* = 0.08). In addition, no association was found between MSI and the MC group.

**Figure 3:**
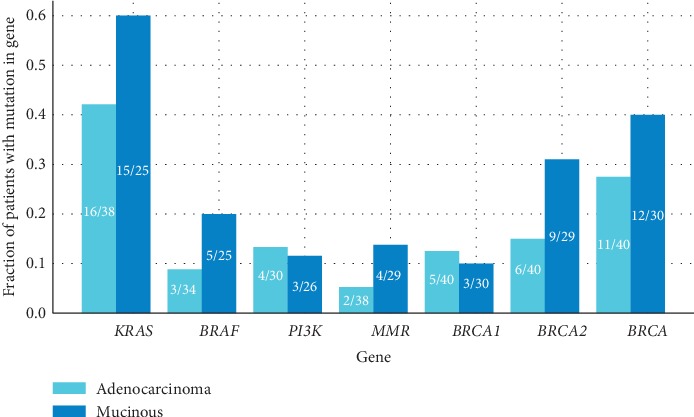
Common mutations in colorectal cancers from patients enrolled to the prospective cohort. The frequency of all genes does not differ in a statistically significant manner between the two groups.

**Figure 4:**
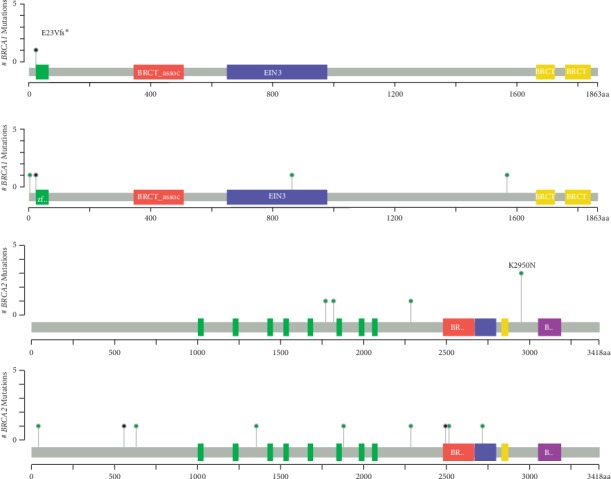
Lollipop plot of identified somatic mutations in *BRCA1* and *BRCA2* in mucinous (MC) and adenocarcinoma (AC) colorectal cancers.

All patients were tested for somatic *BRCA1/2* mutations (Figure 3); among 70 CRC patients, 23 revealed a nonsynonymous *BRCA* mutation (i.e., 32%). Our cohort presents a trend towards a higher frequency of nonsynonymous mutations in either *BRCA1* or *BRCA2* in MC tumors compared to AC tumors, but it is not statistically significant (12/30, 40% of MC group, 11/40, 27% of AC group, *p* value = 0.2705, by chi-squared test). However, when analyzing *BRCA2* mutations separately, we did observe a trend towards a higher frequency of mutations in the MC group (9/29, 31% of MC group, 6/40, 15% of AC group). Additionally, two pathogenic mutations of *BRCA2* were present only in the MC group (c.7480 C > T and c.1670 T > C). Notably, one common mutation (c.8850 G > T) comprised half of the *BRCA2* mutations detected in the AC group. On the *BRCA1* gene, the same pathogenic mutation c.68_69delAG was present in both MC and AC groups. The distribution of mutations along the genes by the cBioPortal mutation mapper tool (https://www.cbioportal.org/mutation_mapper) does not indicate a bias for specific or hotspot locations or domains along the proteins between the two groups (Figure 4).

**Figure 5:**
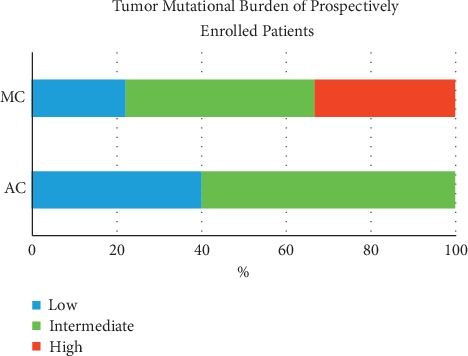
Tumor mutation burden (TMB) of the prospective cohort. Mucinous (MC, *n* = 9) tumors have higher TMB versus adenocarcinoma cancers (AC, *n* = 5), *p*=0.07.

Lastly, since we observed a higher mutation count in MSKCC data for both MC tumors and *BRCA*-mutated tumors, we have decided to perform Tumor Mutation Burden (TMB) analysis in our patients. Fourteen patients were assembled in an attempt to provide a further outlook towards the role of *BRCA* mutations as a marker of high TMB and the relation to MC (MC: 4 *BRCA*-mutated, 5 *BRCA* wild-type (WT); AC: 2 *BRCA*-mutated, 3 *BRCA* WT). Only a single case of MC had MSI. Taking Foundation Medicine cutoff for TMB (low <6, intermediate 6–19, and high >20) [24], we observed that MC tumors are enriched for intermediate-high TMB tumors (Figure 5, Table 5, *p* = 0.07). In addition, *BRCA*-mutated tumors had numerically elevated TMB, in comparison to *BRCA* WT cancers (*p* = 0.14).

## 4. Discussion

In the current work, we have suggested a novel correlation between CRC histology, mutational burden, and *BRCA* mutations.

Our analysis of the MSKCC database detected a statistically significant higher incidence of *BRCA* mutations in the MC group as listed above (19/128 MC 14.8%, 30/725 AC 4.1%, *p* value <0.001, by chi-squared test). Additionally, average mutation counts in tumors with MC histology were higher compared with the AC group (24.8 and 8.9, respectively, *p* value <0.001). Our analysis might shed a light into the relationship between *BRCA* mutations and high mutation counts, since the mutated *BRCA* group has shown higher mutation counts compared with the *BRCA* WT group (average of 59.4 versus 9.4, respectively, *p* value <0.001).

Furthermore, we demonstrated two distinct groups of tumors with *BRCA* mutations: a high-mutation-count group with both mucinous histology and high MSI and a low-mutation-count group with both adenocarcinoma histology and low MSI score.

This finding can be explained by the well-known association between mucinous histology and MSI, suggesting MSI as a reasonable explanation for the high mutation counts in the MC group. Nevertheless, our analysis further revealed a small group of *BRCA*-mutated tumors with high mutation counts and a low MSI score (Figure 1(b)), possibly implicating *BRCA* as an independent predictor of high mutation count.

It is interesting to ponder what characterizes these different subgroups and what causes the high mutation count in each case. To further study what variables determine the mutation count, we constructed a linear regression model demonstrating that nearly 0.4 of the variance in the mutation count between the different patients with *BRCA* mutations could be explained using a small number of variables (Table 2, Table 3). Some of the variables were not statistically significantly linked to the mutation count within the regression model, indicating additional correlations between variables within the model which explain the same variance in the mutation count. The strongest predictor of mutation counts was mucinous histology, independently of other variables, possibly suggesting that this feature determines the mutation count in patients with *BRCA* mutations.

Since the linear regression model indicated that mucinous histology, and not MSI, is the best predictor of mutation counts, it is possible that the *BRCA*-mutated low-MSI, high-mutation-count group is associated with mucinous histology. Our data also correlates with a previous report by Ciriello et al. [26], who characterized a subset of ultramutated CRC with an altered double-strand break repair mechanism. Notably, >50% of these tumors had somatic mutations in *BRCA1/2* genes. However, a further study should be done to validate and establish the existence of this specific subgroup.

With the intention to robustly establish the link between MC histology and *BRCA* mutations, we tested a cohort of AC and MC patients with similar background features in our medical center (Figure 3). Unfortunately, we could not reestablish the statistically significant link between *BRCA* mutations and the MC group (12/30, 40% of MC group and 11/40, 27% of AC group were *BRCA*-mutated).

Notably, even mutations that are known to be found in significantly higher incidence in MC tumors such as *KRAS*, *BRAF*, and *PI3K* were not seen in our cohort, prompting a suspicion that the lack of association is related to limitations of this specific cohort itself, and may explain our failure to reestablish the link between *BRCA* mutations and MC histology. Indeed, this analysis was performed on a limited sample size and with a broad definition of MC histology. This broad definition was linked to the variance between observers and to the MC WHO criteria, which are based on the evaluated amount of mucin, a component that is difficult to define accurately. However, we observed a trend towards a higher frequency of *BRCA2* mutations in the MC group (9/29, 31% of MC group, 6/40, 15% of AC group).

Lastly, since we observed a higher mutation count in MSKCC data, we have decided to further investigate the implications of this finding and to reestablish it in our local cohort. To link mutation count and TMB, we relied on a previous method described by Chalmers et al. [24], where mutation count was divided by the estimated exome sample size of 38 Mb to calculate mutation count per MB. Mutation count per MB was found equivalent to TMB per MB as both represent the total number of mutations counted divided by the size of the coding region of the targeted territory.

Later, TMB analysis was performed in a prospective cohort (Figure 5, Table 5). We observed that MC tumors are enriched for intermediate-high TMB tumors (Figure 5, *p* = 0.07). A study by Naseem et al. [27] may hint at the importance of this finding; this impressive study presented 6396 CRC tumor samples tested with next-generation sequencing for pathogenic mutations, MSI and TMB. *BRCA* pathogenic mutations were detected in 1.1% (*n* = 72) of tumors, while *BRCA2* in 2.8% (*n* = 179). *BRCA1/2*mutations were associated with higher TMB in all CRCs, including MSI-H and MSS cases (*p* < 0.001). Among MSS cases with *POLE* wild-type status, *BRCA1* (*p* = 0.0269) and *BRCA2* (*p* = 0.0151) mutations were associated with high TMB and combining both *BRCA1/2* mutations led to an even higher TMB (3.6%; *p* = 0.001). *BRCA1/2* mutations are more frequent in MSI-H and independently associated with higher TMB, pathogenic *POLE* mutations, and right-sided tumors in MSI-H CRCs [27]. Potentially, the findings may indicate that the lack of a functioning DNA repair mechanism might be the driver for a higher-mutation load or alternatively that the mutations in the *BRCA* genes themselves are passenger mutations due to the overall increased mutations load.

**Figure 6:**
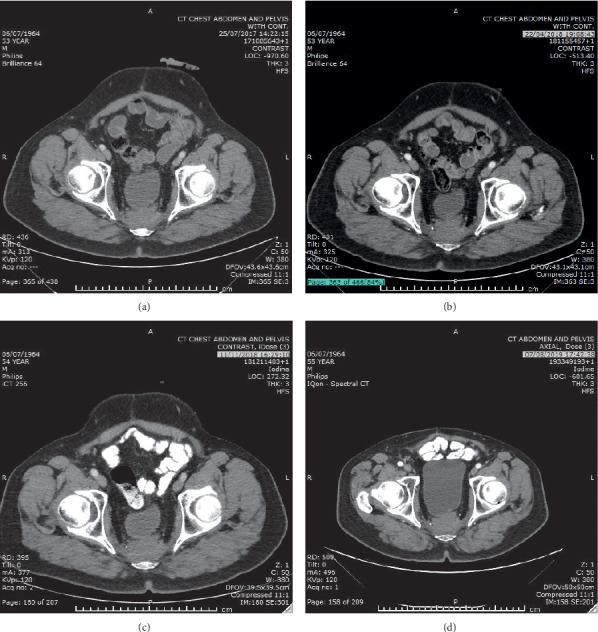
Stability of presacral CRC metastatic lesion on PARP inhibitor therapy. Axial computed tomography scans during treatment are provided: (a) July 2017, (b) April 2018, (c) November 2018, and (d) August 2019.

An intriguing question might be “what is the further impact of our findings on the evaluation of CRC patients of Jewish-Ashkenazi ancestry, for whom the incidence of germline *BRCA* and Lynch syndrome mutations are higher [28]?” It is important to emphasize that genetic testing for germline mutations involves important ramifications regarding the genetic counseling needed for descendants and the potential cascade testing. Thus, testing for germline mutations warrants patients' consent and understanding. Moreover, we tried to utilize PARP inhibition approach in one of the patients in our cohort, as PARP inhibition is synthetically lethal in *BRCA*-deficient tumors (FDA approved for ovarian, pancreatic, and breast tumors with *BRCA1/2* mutation [5, 25, 29, 30]). The patient was a 53-year-old male with rectal adenocarcinoma (mismatch repair proficient, KRAS and BRAF wild-type) with pelvic and lung metastases. He underwent somatic tumor analysis that showed pathogenic *BRCA1* mutation (c.68_69delAG), later proved to be germline. Following achievement of maximal response to first-line chemotherapy with FOLFOX and anti-EGFR antibody (Panitumumab), the patient started Veliparib (PARP inhibitor, kindly provided by AbbVie) on July 2017. The treatment was well tolerated on 300 mg BID and the patient remained with stable disease (Figure 6) for almost 23 months (June 2019), when new mediastinal and pulmonary lesions appeared. As represented here, PARP inhibitors might serve as a potential future therapeutic approach in *BRCA*-mutated CRC, especially for challenging MC patients.

In addition, emerging evidence suggests that *BRCA1* mutation may even influence the survival outcomes among metastatic CRC patients treated with Oxaliplatin or Irinotecan-based regimens [31].

Taken together, this data imply that *BRCA1/2* and MC histology may serve as a potential surrogate marker for tumors with higher TMB. This “low-tech” biomarker can increase the number of patients who may benefit from novel treatment strategies based on immunotherapy for TMB-high tumors [28]. Further studies are required to elucidate the real-world value of TMB analysis in MC colorectal cancer with or without *BRCA1/2* mutation.

## Figures and Tables

**Table 1:** Investigation of mutation count characteristics in metastatic colorectal cancer database (MSKCC, Cancer Cell 2018). Significant **p** values are marked in bold.

Parameters	Test for mutation	*p* value for mutation count	Corrected *p* value for mutation count
Age at diagnosis	Spearman	**p** value <0.05	**p** value <0.05
Sex	Mann–Whitney	*p* value >0.05	*p* value >0.1
First site of metastasis	Kruskal–Wallis	*pvalue >0.05*	*pvalue >0.1*
Fraction of genome altered	Spearman	**p** value <0.05	**p** value <0.1
Tumor sample histology	Mann–Whitney	**p** value <0.05	**p** value <0.05
Stage at diagnosis	Kruskal–Wallis	**p** value <0.05	**p** value <0.05
Primary tumor location	Mann–Whitney	**p** value <0.05	**p** value <0.05
Primary tumor site	Kruskal–Wallis	**p** value <0.05	**p** value <0.05
MSI score	Spearman	**p** value <0.05	**p** value <0.05
BRCA mutations	Mann–Whitney	**p** value <0.05	**p** value <0.05

Additional analyses			
BRCA mutations and tumor sample histology	Chi-square	**p** value <0.05	**p** value <0.05

Stratified mutation count analysis			
BRCA mutations among MC	Mann–Whitney	**p** value <0.05	**p** value <0.05
BRCA mutations among AC	Mann–Whitney	**p** value <0.05	**p** value <0.05
Tumor sample histology and *BRCA* mutations	Mann–Whitney	**p** value <0.05	**p** value <0.05
Tumor sample histology and *BRCA* WT	Mann–Whitney	**p** value <0.05	**p** value <0.05

^*∗*^Threshold for significance after correction for multiple hypotheses was 0.1.

**Table 2:** Association of different variables and mutation count involved in discrimination between the *BRCA*-mutated/high-mutation-count group and the *BRCA*-mutated/low-mutation-count group. Significant *p* values are marked in bold.

Parameters	Test for mutation	*p* value for mutation count	Corrected *p* value for mutation count	Test for *BRCA*_mut_group	*p* value for *BRCA*_mut_group	Corrected *p* value for *BRCA*_mut_group
Age at diagnosis	Spearman	*p* value >0.05	*p* value >0.1	Mann–Whitney	*p* value >0.05	*p* value >0.1

Sex	Mann–Whitney	*p* value >0.05	*p* value >0.1	Chi-square	*p* value >0.05	*p* value >0.1

First site of metastasis	Kruskal–Wallis	*p* value >0.05	*p* value >0.1	Fisher's exact test	*p* value >0.05	*p* value >0.1

Fraction of genome altered	Spearman	**p** *value <0.05*	**p** *value <0.05*	Wilcoxon	**p** *value <0.05*	**p** *value <0.05*

Tumor sample histology	Mann–Whitney	**p** *value <0.05*	**p** *value <0.05*	Chi-square	**p** *value <0.05*	**p** *value <0.1*

Stage at diagnosis	Kruskal–Wallis	**p** *value <0.05*	**p** *value <0.05*	Fisher's exact test	**p** *value <0.05*	**p** *value <0.05*

Primary tumor location	Mann–Whitney	**p** *value <0.05*	*p* value >0.1	Chi-square	**p** *value <0.05*	*p* value >0.1

MSI score	Spearman	**p** *value <0.05*	**p** *value <0.05*	Mann–Whitney	**p** *value <0.05*	**p** *value <0.05*

^*∗*^Threshold for significance after correction for multiple hypotheses was 0.1.

**Table 3:** Linear regression model for the mutation count in the MSKCC database.

Parameters	Estimate	Std error	*t* value	Pr(>|*t*|)
Intercept	38.9395	31.4677	1.237	0.22332
Fraction of genome altered	−134.5028	50.6571	−2.655	0.01142
Tumor sample, mucinous	51.2031	18.705	2.737	0.00928
MSI score	−0.5486	0.6408	−0.856	0.39719
Primary tumor location, right	35.694	17.7	2.017	0.05066
Stage at diagnosis II	−6.9323	32.7837	−0.211	0.83363
Stage at diagnosis III	9.7213	30.1277	0.323	0.74867
Stage at diagnosis IV	−5.253	28.6696	−0.183	0.85557

Multiple *R*-squared	0.3829			
Adjusted *R*-squared	0.2721			
*F*-statistic	3.457 on 7 and 39 DF			
*p* value	0.005653			

**Table 4:** Prospective cohort patients' characteristics.

	Adenocarcinoma (*n* = 40)	Mucinous (*n* = 30)
Sex		
Female	23 (57.5%)	12 (40.0%)
Male	17 (42.5%)	18 (60.0%)

Age at diagnosis		
Mean (SD)	59.1 (13.8)	60.2 (14.7)
Mean (min, max)	60.0 (20.0, 86.0)	63.5 (22.0, 78.0)

Ethnic origin		
Arab	9 (22.5%)	6 (20.0%)
Jewish-Ashkenazi	16 (40.0%)	15 (50.0%)
Jewish-non-Ashkenazi	12 (30.0%)	7 (23.3%)
Missing	3 (7.5%)	2 (6.7%)

Family breast history		
No	33 (82.5%)	23 (76.7%)
Yes	7 (17.5%)	7.(23%)

Previous malignancy		
No	36 (90.0%)	26 (86.7%)
Yes	4 (10.0%)	4 (13.3%)

Stage at diagnosis		
I	3 (7.5%)	0 (0%)
II	4 (10.0%)	5 (16 7%)
III	10 (25.0%)	13 (43 3%)
IV	23 (57.5%)	11 (36 7%)
Missing	0.(0%)	1 (3.3%)

Primary tumor site		
Left	25 (65.5%)	18 (60.0%)
Right	10.(25.0%)	11 (36.7%)
Missing	5 (12.5%)	1 (3.3%)

Metastases primary site		
Abdomen	4 (10.0%)	6 (20.0%)
Distant	4 (10.0%)	3 (10.0%)
Liver	22 (55.0%)	6 (20.0%)
Pelvis	3 (7.5%)	4 (13.3%)
Missing	7 (17.5%)	11 (36.7%)

Surgery		
No	4 (10.0%)	6 (20.0%)
Yes	34 (85.0%)	23 (76.7%)
Missing	2 (5.0%)	1 (3.3%)

Adjuvant treatment		
FOLFOX	6 (15.0%)	7 (23.3%)
None	22 (55.0%)	12 (40.0%)
Oxaliplatin, Fluorouracil	0 (0%)	1 (3.3%)
XELODA	1 (2.5%)	(2.6.7%)
XELOX	3 (7.5%)	7 (23.3%)
Missing	8 (20.0%)	1 (3.3%)

**Table 5:** Tumor mutation burden (TMB) of the prospective cohort. Mucinous (MC, *n* = 9) tumors have higher TMB versus adenocarcinoma cancers (AC, *n* = 5), *p*=0.07.

	Number of patients	Average TMB (mut/megabase)	High TMB^*∗*^	Low TMB^*∗*^
Mucinous	9	43.07	3, 33%	6, 66%
Mucinous *BRCA* mut	4	84.03	2, 50%	2, 50%
Mucinous *BRCA* WT	5	10.3	1, 20%	4, 80%

Adenocarcinoma	5	5.9	0, 0%	5, 100%
Adenocarcinoma *BRCA* mut	2	4.91	0, 0%	2, 100%
Adenocarcinoma *BRCA* WT	3	6.56	0, 0%	3, 100%

^*∗*^Number of patients, % of patients with high/intermediate/low TMB. ^*∗∗*^High TMB using standard cutoff of >20.

## Data Availability

The data used to support the findings of this study are available from the corresponding author upon request.

## Supplementary Materials

Supplementary Materials Anonymized individual patient data used to support the findings of this study are included within Supplementary Materials.
