# Corrigendum to “Mucinous Histology, *BRCA1/2* Mutations, and Elevated Tumor Mutational Burden in Colorectal Cancer”

**DOI:** 10.1155/2020/6725209

**Published:** 2020-09-11

**Authors:** Noa Harpaz, Yair Eli Gatt, Roy Zvi Granit, Hila Fruchtman, Ayala Hubert, Albert Grinshpun

**Affiliations:** ^1^Sharett Institute of Oncology, Hadassah-Hebrew University Medical Center, Jerusalem, Israel; ^2^Department of Microbiology and Molecular Genetics, Institute for Medical Research Israel-Canada, Faculty of Medicine, The Hebrew University of Jerusalem, Jerusalem, Israel; ^3^Radiology Department, Hadassah-Hebrew University Medical Center, Jerusalem, Israel

In the article titled “Mucinous Histology, BRCA1/2 Mutations, and Elevated Tumor Mutational Burden in Colorectal Cancer” [1], some information was omitted in the Acknowledgments section, which should be corrected as follows:
